# Individual dietary specialization in a generalist predator: A stable isotope analysis of urban and rural red foxes

**DOI:** 10.1002/ece3.6584

**Published:** 2020-07-17

**Authors:** Carolin Scholz, Jasmin Firozpoor, Stephanie Kramer‐Schadt, Pierre Gras, Christoph Schulze, Sophia E. Kimmig, Christian C. Voigt, Sylvia Ortmann

**Affiliations:** ^1^ Department of Ecological Dynamics Leibniz Institute for Zoo and Wildlife Research Berlin Germany; ^2^ Department of Evolutionary Ecology Leibniz Institute for Zoo and Wildlife Research Berlin Germany; ^3^ Institute of Ecology Technische Universität Berlin Berlin Germany; ^4^ Berlin‐Brandenburg Institute of Advanced Biodiversity Research (BBIB) Berlin Germany; ^5^ State Laboratory Berlin Brandenburg (LLBB) Frankfurt (Oder) Germany; ^6^ Institute of Biology Freie Universität Berlin Berlin Germany

**Keywords:** anthropogenic, carnivore, diet, red fox, specialization, stable isotope, urbanization, *Vulpes vulpes*

## Abstract

Some carnivores are known to survive well in urban habitats, yet the underlying behavioral tactics are poorly understood. One likely explanation for the success in urban habitats might be that carnivores are generalist consumers. However, urban populations of carnivores could as well consist of specialist feeders. Here, we compared the isotopic specialization of red foxes in urban and rural environments, using both a population and an individual level perspective. We measured stable isotope ratios in increments of red fox whiskers and potential food sources. Our results reveal that red foxes have a broad isotopic dietary niche and a large variation in resource use. Despite this large variation, we found significant differences between the variance of the urban and rural population for δ^13^C as well as δ^15^N values, suggesting a habitat‐specific foraging behavior. Although urban regions are more heterogeneous regarding land cover (based on the Shannon index) than rural regions, the dietary range of urban foxes was smaller compared with that of rural conspecifics. Moreover, the higher δ^13^C values and lower δ^15^N values of urban foxes suggest a relatively high input of anthropogenic food sources. The diet of most individuals remained largely constant over a longer period. The low intraindividual variability of urban and rural red foxes suggests a relatively constant proportion of food items consumed by individuals. Urban and rural foxes utilized a small proportion of the potentially available isotopic dietary niche as indicated by the low within‐individual variation compared to the between‐individual variation. We conclude that generalist fox populations consist of individual food specialists in urban and rural populations at least over those periods covered by our study.

## Graphical abstract

We compared the isotopic specialization of red foxes in urban and rural environments, using both a population and an individual level perspective. We found significant differences between the variance of the urban and rural population for δ13C as well as δ15N values, suggesting a habitat‐specific foraging behavior. Furthermore, generalist fox populations consist of individual food specialists in urban and rural populations.

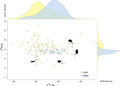

## 1 INTRODUCTION

Our environment is subject to constant anthropogenic influence, with urbanization being among the most outstanding example of habitat transformation for wildlife species (Grimm et al., 2008; Magle, Hunt, Vernon, & Crooks, 2012), including vital food sources. This transformation, directly and indirectly, affects wildlife at the individual (Newsome, Garbe, Wilson, & Gehrt, 2015), population (Davison, Huck, Delahay, & Roper, 2009), and community level (Prange & Gehrt, 2004). Although urbanization is one of the major threats to global biodiversity (McKinney, 2002), cities are growing, expanding into previously natural habitats, and becoming increasingly populated by a large number of wildlife species (McKinney, 2008). Many of them have adjusted their behavior to these human‐dominated, novel habitats others have been suppressed (Bateman & Fleming, 2012).

The red fox (*Vulpes vulpes*) is one example for a successful synanthropic species and known for its wide distribution, its flexibility in habitat use, feeding, social organization, and thus for its high adaptability (Macdonald, 2011). Successful breeding and flourishing fox populations in urban areas have been recorded in large cities and metropolitan areas, such as London (Page, 1981), Bristol (Harris, 1981), Toronto (Adkins & Stott, 1998), Zurich (Hofer et al., 2000), and Berlin (Börner & Schneider, 2009), among many others.

Access to food as vital bottom‐up factor plays a key role in the success of animals, as it influences body condition and thus reproductive success and fitness. The utilization of a wide range of food resources can be advantageous in dynamic habitats with constantly changing food availability. Previous studies on red fox diet showed that its feeding behavior is highly flexible, spanning multiple trophic levels from berries to insects to small mammals (e.g., Calisti, Ciampalini, Lovari, & Lucherini, 1990; Harris, 1981; Leckie, Thirgood, May, & Redpath, 1998; Macdonald, 2011). Usually, the most abundant and most accessible food source is used, which varies with resource availability (Calisti et al., 1990; Cavallini & Volpi, 1996; Ferrari, 1995; Leckie et al., 1998). In cities, food quality and obtainability are strongly influenced by humans (Baker, Funk, Harris, & White, 2000), and anthropogenic food, besides, seems to play a major role in urban red fox diet (Contesse, Hegglin, Gloor, Bontadina, & Deplazes, 2004; Doncaster, Dickman, & Macdonald, 1990; Harris, 1981; Saunders, White, Harris, & Rayner, 1993).

**Figure 1:**
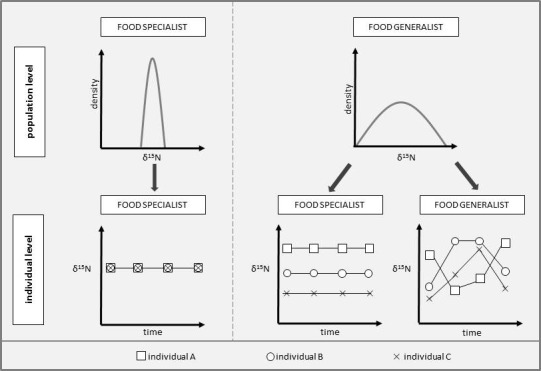
Conceptual diagram of how individuals can contribute to the population's dietary niche. Specialized populations consist of specialized individuals which all consume certain resources (left). Therefore, their total dietary niche represents a small dietary variation within and between individuals. In contrast, generalistic populations can consist either of specialized or generalistic individuals (right). In this case, specialized individuals show small dietary variation within individuals, but a large dietary variation between individuals leads to a broad overall resource spectrum and dietary variation at the population level. If individuals of a generalistic population forage generalistic then those are characterized by a large within‐individual dietary variation

In our context, at the individual level, foraging specialists are individuals whose dietary niche (which represents the extent of the food spectrum) is smaller than the total dietary niche width of the population. Foraging generalists, in contrast, are individuals varying widely in their resource use and therefore represent the whole niche of the associated population (Bolnick, Yang, Fordyce, Davis, & Svanback, 2002). Many species are commonly described as generalist foragers. However, generalist species can also enclose specialized individuals (see Figure 1), each using only a small part of the entire feeding spectrum. The total dietary niche width of a species represents the sum of consumed prey species within the population and therefore population variation (Bolnick et al., 2002).

The diet of red foxes has been widely studied on a population level (Díaz‐Ruiz et al., 2013; Soe et al., 2017). However, knowledge of individual feeding tactics (generalized or specialized food selection) and temporal tactic stability (in the sense of a constant tactic over time) is still limited. For red foxes, we are currently unaware of any study about individual feeding tactics using repeated measurements of the same individual, that is, the level of individual dietary specialization and whether those differ among urban and rural populations. A potentially individual dietary specialization could have complex consequences (Araújo, Bolnick, & Layman, 2011). It is an important component in trophic interactions and food web structures, it enhances our understanding of selective pressure on prey and it has implications in the transmission and of parasites and pathogens (Bolnick et al., 2011). Furthermore, there is growing evidence that individual variation in resource use has implications for intra‐ and interspecific competition and population dynamics. Therefore, understanding the causes and importance of individual dietary specialization is a major goal in animal ecology (Araújo et al., 2011).

Stable isotopes have become a useful tool to study dietary niches, representing the trophic ecology of organisms (e. g., Layman, Arrington, Montaña, & Post, 2007). The power of the stable isotope method stems from the fact that isotope values measured of consumer tissues are related to the corresponding consumers’ diet (DeNiro & Epstein, 1978, 1981). Stable isotope analysis can be used to investigate resource use patterns across different organization levels and over different time scales, depending on the consumer tissue used. The analyses of carbon and nitrogen stable isotopes have also been successfully used as a proxy for diet specialization in predators (Anderson et al., 2009; Cherel, Hobson, Guinet, & Vanpe, 2007; Lavin, Van Deelen, Brown, Warner, & Ambrose, 2003; Matich, Heithaus, & Layman, 2011; Newsome, Ralls, Job, Fogel, & Cypher, 2010; Newsome et al., 2009; Voigt, Krofel, Menges, Wachter, & Melzheimer, 2018; Voigt et al., 2014; Woo, Elliott, Davidson, Gaston, & Davoren, 2008). Stable carbon isotopes reflect the baseline producers or the habitat whereas nitrogen isotopes are primarily influenced by the trophic position of the species. The stable isotope signatures of tissue generally reflect the diet composition during the period of tissue synthesis (Bearhop, Waldron, Votier, & Furness, 2002; Hobson & Clark, 1992) and the variance of isotopic values within these tissues is used as a measure of niche width (Bearhop, Adams, Waldron, Fuller, & Macleod, 2004). The more different prey species with different isotopic signatures are consumed the more variable is the isotopic signature, whereas dietary specialists focusing on a few prey items show a small variance in the isotopic signature of their tissues and therefore a narrower niche (Bearhop et al., 2004). Besides, individuals of populations consuming widely differing proportions of each prey over time will tend to show less variation in the C and N ratios than what is expected for constant proportions of each prey and therefore high evenness in the diet (Bearhop et al., 2004). However, feeding on many prey species may not necessarily lead to large isotopic variance in the consumer tissue, if prey species are isotopically similar (Martínez del Rio, Wolf, Carleton, & Gannes, 2009) or the amount of the consumed tissue varies. Finally, stable isotope analysis allows us to characterize the inter‐ and intraindividual variation of the diet. This could be an effective way to investigate dietary specialization, because the variance between and within individuals can then be compared to the associated population.

In this study, we used stable isotopic ratios of red fox whiskers (vibrissae) to quantify and compare the isotopic dietary niche width and feeding tactics of urban and rural red foxes at (I) the population level (single measurements of 119 red foxes) and (II) the individual level (each time 5 increments of 32 individuals reflecting five times 11 days). For assessing the individual isotopic specialization, we used carbon (δ^13^C) and nitrogen (δ^15^N) stable isotope ratios of vibrissae increments, which provided us with a temporally continuous isotopic record within the same individual. To delineate the feeding habits of red foxes, we compared stable isotope ratios of red foxes with those of potential food items using Bayesian isotope mixing models.

On a population level, we hypothesize that isotopic signatures of urban red foxes differ noticeably from rural conspecifics. Taking into account previous studies on the feeding ecology of red foxes, we assume that urban populations consume a large proportion of anthropogenic food sources (e.g., food scraps, garbage, and pet food), consisting of a mixture of different food items that are isotopically contrasting with natural food sources. Therefore, we predict a smaller isotopic niche for urban foxes, since cities have a relatively constant supply of anthropogenic food throughout space and time. In contrast, the abundance and availability of food resources in rural areas are habitat‐dependent and variable over time and space, which should be reflected in a larger dietary (isotopic) niche compared to urban foxes. Assuming that foxes nevertheless concentrate within their individual range on the most available and easiest to obtain food item, this should take up a large proportion of the fox diet and thus result in low variability in isotopic signatures over time in rural and urban foxes (individual level). Therefore, both rural and urban red fox individuals follow an (optional) specialized feeding tactic, even though foxes are a generalistic species at the population level.

## 2 MATERIALS AND METHODS

### 2.1. Study area and sample collection

2.1

**Figure 2:**
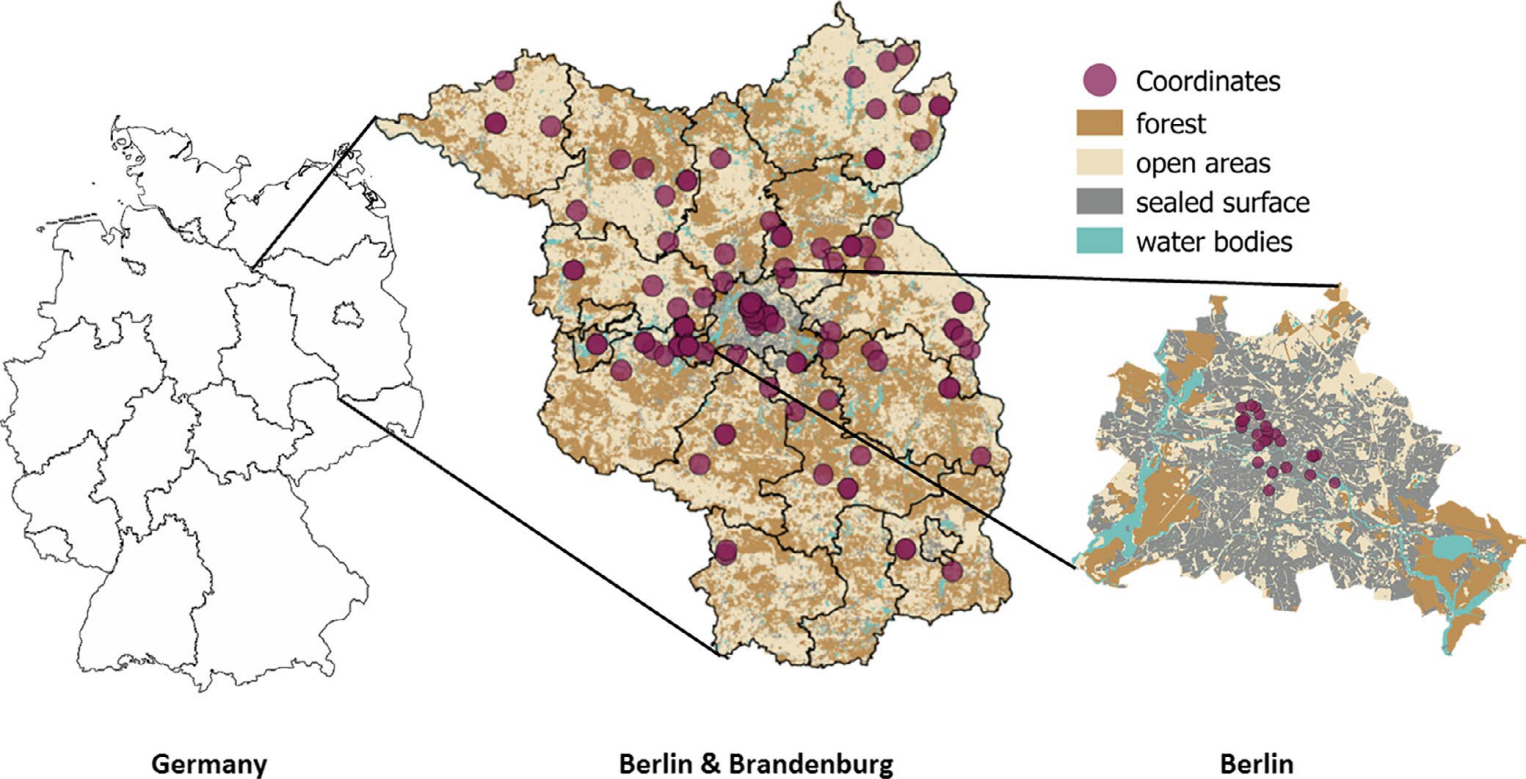
Location of the study area in northeastern Germany. The Berlin and Brandenburg map shows the landscape composition as well as the distribution of red fox samples (*n* = 119)

The study was conducted in Berlin and Brandenburg in the northeastern part of Germany (Figure 2). Both districts together cover an area of 30.371 km^2^ with a maximum diagonal extension of 291 km. Berlin as capital is characterized by highly urbanized areas, especially in the city center, whereas the surrounding federal state of Brandenburg is characterized by rural areas composed of small forests mostly embedded in agricultural landscapes. In the metropolitan area of Berlin, the density of humans increases toward the city center, forming a suburban area connecting the rural regions of Brandenburg and the highly urbanized areas of Berlin gradually.

Red foxes are found all over the study area, populating rural areas as well as highly urbanized regions. In cooperation with the state laboratory Berlin‐Brandenburg (LLBB), we collected a total of 119 whisker samples from dead red foxes originating from urban and rural environments. These samples stem from foxes that were either involved in accidents, were hunted or died of natural causes in the years of 2016 and 2017. Samples of urban and rural foxes were collected throughout the year with fewer data in spring and beginning summer.

To assign each fox sample to the “rural” or “urban” category, we calculated the percentage of imperviousness within a 1 km radius (reflecting approximately the size of a generously red fox home range) of each location of death. For this, we used a COPERNICUS imperviousness raster map of 2012 with 20 m resolution (http://land.copernicus.eu/pan‐european/high‐resolution‐layers/imperviousness/%20imperviousness‐2012/%20view"\h) and extracted the mean of all raster cells within the buffer. Locations having a degree of imperviousness lower than 25% were categorized as “rural,” all other locations (≥25%) were assigned to the category “urban.” In the end, 85 of the individuals were assigned to the category “rural” and 34 to “urban.” Imperviousness is considered to be a suitable proxy for urbanization because it is also associated with factors such as human population density, light pollution, traffic, and noise (Kuechly et al., 2012; Kasanko et al., 2006).

Besides, we characterized the heterogeneity of the landscape by using a land‐use map of Berlin (https://fbinter.stadt‐berlin.de/fb/index.jsp; Umweltatlas Berlin/Stadtstruktur‐Flächentypen differenziert 2015 (Umweltatlas)) and Brandenburg (https://lfu.brandenburg.de/cms/detail.php/bb1.c.359429.de). Since each map has its resolution regarding the land use categories, or names them partially differently, we have assigned all land cover types to the following to have a common basis. Nine land use categories were used: agriculture, forest, grassland, open areas, ruderal areas, shrubland, sealed surface, water bodies, and others. As before, landscape diversity (Shannon diversity index of the nine land use categories) was calculated within a 1 km zone around each sample location (see Appendix).

To understand diet composition, we collected potential food sources as reference values for our analysis to confirm the availability of typical food resources over the entire study area (rural and urban) and to see whether the stable isotope values of food resources vary greatly between the contrasting habitats. Since we were mainly interested in breaking down nutritional tactics and their stability over time instead of exact resource use, the analysis of food items served more as control and at the same time nicely estimates the position within the food niche. Inexperienced readers thus get a direct impression of the potential food as well as its position and can more easily follow our reasoning. We also considered adding anthropogenic food items to the food item analysis, but since these can be very diverse and are often a mixture of different resources, we consciously decided against it. Based on literature research about diet composition of red foxes in our study region and availability of food items, we chose seven potential food sources at the family level with the main focus on covering different trophic levels: dor beetle (*Geotrupidae*), earthworm (*Lumbricidae*), grasshopper (*Orthoptera*), land snail (*Helicidae*), land slug (*Limacidae*), house mouse (*Muridae*), and bramble (*Rosaceae*) (e.g., Calisti et al., 1990; Drygala, Werner, & Zoller, 2013; Harris, 1981; Leckie et al., 1998; Macdonald, 2011). Ten samples for each food category were collected in six different locations in Berlin and seven sites in Brandenburg during July and October 2017 (see Table 1). All samples were frozen on the day of collection and stored at −80°C until analysis.

**Table 1:** Mean δ^13^C or δ^15^N values and standard error of the mean (SE) of red fox food items collected in Berlin and Brandenburg, Germany. δ^13^C_cor_ values are corrected for a TEF of 4.31‰ and δ^15^N_cor_ for a TEF of 3.05‰

			**δ^13^C (**‰**)**			**δ^15^N (**‰**)**		
**Common name**	**Family**	***n***	**Mean**	***SE***	**δ^13^C_cor_**	**Mean**	***SE***	**δ^15^N_cor_**
Earthworm^a^	Lumbricidae	10	−25.7	0.3	−21.4	2.0	0.9	5.0
Dor beetle	Geotrupidae	10	−24.8	0.4	−20.5	3.8	0.7	6.9
Grasshopper	Orthoptera	10	−28.3	0.2	−23.9	2.7	1.2	5.8
Land slug^a^	Limacidae	10	−25.9	1.0	−21.6	2.2	1.0	5.3
Land snail^a^	Helicidae	10	−26.4	0.6	−22.1	1.7	0.9	4.7
Bramble^b^	Rosaceae	10	−30.1	0.5	−25.8	−2.3	1.7	0.8
House mouse	Muridae	10	−25.0	0.4	−20.7	6.2	0.8	9.2

^a^ Were pooled together.

^b^ was removed in further analyses.

### 2.2. Sample preparation and analysis

2.2

We used whiskers because this body product has proved useful to delineate temporal changes in the isotopic data of mammals (Darimont & Reimchen, 2002; Newsome et al., 2009, 2010; Voigt et al., 2018). Here, we assume that the whiskers of red foxes grew at a constant rate (Robertson, McDonald, Delahay, Kelly, & Bearhop, 2013; Mutirwara, Radloff, & Codron, 2018). We selected whiskers with an average length of 4 cm because they were sufficiently long and thick to guarantee repeated measurements of stable isotopes in whisker increments. Single whiskers put into a 1:2 methanol:trichlormethan solution in plastic tubes to cleanse them from surface contaminants. After shaking for 24 hr, the liquid was removed and the clean whiskers were dried in an oven [*Heraeus Function Lab*] at 50°C for an additional 24 hr.

The metabolic rates between different organ tissues differ. Therefore, also the turnover rates and enrichment of stable isotopes differ as well (Hobson & Clark, 1992; Tieszen, Boutton, Tesdahl, & Slade, 1983). δ^13^C and δ^15^N values may vary systematically between an animal's tissues and its food, an offset called trophic enrichment factor (TEF; Parnell, Inger, Bearhop, & Jackson, 2010; Tibbets, Wheeless, & del Rio, 2007). Since TEF may vary across taxa, it is important to establish taxon‐specific TEF for the specific study species. A study on stable carbon and nitrogen isotopic fractionation between diet and tissue of captive red foxes determined values for blood, fur, liver, and muscle (Roth & Hobson, 2000). Since the TEF and the growth rate of whiskers in red foxes are still unknown, we assumed that foxes show values comparable to other canids. Thus, we used data from a closely related species, the wolf (*Canis lupus),* as a reference (McLaren, Crawshaw, & Patterson, 2015). For wolves, a TEF δ value for carbon of 4.31‰, for nitrogen of 3.05‰ and an average growth rate of 0.43 mm/day were reported.

To access the diet niche of red foxes at the population level, we selected 119 foxes (males and females) and cut the basal 5 mm increment of the whiskers, using a scalpel. Assuming a growth rate of 0.43 mm/day (McLaren et al., 2015), the sample represents food consumption of the last 11 days. To determine the diet niche of urban and rural red foxes at the individual level, we chose 19 adult urban foxes and 13 adult rural foxes (males and females) from these 119 individuals. We focused on adult foxes (>1 year), as they tend to remain stationary within a defined area throughout their lives. We cut further 5mm long increments of whiskers at 10, 15, 20, and 25 mm from the root, reflecting approximately 55 days in steps of 11 days each. All segments were weighed with an analytical microbalance (0.5 ± 0.1 mg), placed in tin capsules [*OEA Labs*, 6 mm × 4 mm], folded tightly, sealed and gently compacted into small cubes. The cubes were placed in a clean 96 position plastic culture tray [ELISA plate].

All food samples were defrosted and washed with distilled water. Indigestible parts such as chitin shells from beetles and grasshoppers or shells from land snails were removed. A small representative piece of each sample was cut, placed into a 2 ml tube and dried at 50°C for 48 hr [Heraeus Function Lab]. Afterward, 110 ml of a 1:2 methanol:trichlormethan solution was added and the fat was extracted using a rapid extraction system [C. Gerhardt GmbH, SOXTHERM]. For extraction, sample solutions were boiled at 140°C for 30 min, distilled and readded to the samples. We ran four extraction cycles of 25 min each. After extraction, samples were dried again at 50°C for 24 hr. Finally, food samples were weighed and loaded into tin capsules following the protocol of whisker samples described above. Samples were combusted and analyzed using a peripheral elemental analyzer [*Flash EA 1112 Series*, *Thermo Fisher, Bremen, Germany*] coupled to a stable isotope ratio mass spectrometer [*Delta V Advantage, Thermo Fisher*] in continuous flow. For the calculation of isotope ratios, laboratory reference materials were used. The isotopic values for carbon are expressed in delta notation (in ‰ units) relative to Vienna Pee Dee Belemnite (VPDB). For the stable nitrogen isotopes, atmospheric nitrogen was used as the standard.

### 2.3 Data analysis

All data analyses were performed with R Studio in R version 3.5.0 (R Core Team 2018).

#### 2.3.1 Population level

We estimated and plotted the isotopic dietary niche metrics of urban and rural foxes based on stable isotope ratios of single individuals using Stable Isotope Bayesian Ellipses in R (SIBER package: Jackson, Inger, Parnell, & Bearhop, 2011). The SIBER package is used to compare isotopic niches across communities by analyzing the isotopic distribution of consumer tissues. The metrics of SIBER take the uncertainty in the sampled data into account and naturally incorporate errors arising from the sampling process, propagating it through to the derived metrics. Therefore, calculated ellipses are unbiased to sample size and allow robust comparison among datasets comprising different sample sizes (Jackson et al., 2011). Thus, the standard ellipse area (SEA) corrected for small sample size (SEAc) represents the trophic niche breadth. We calculated the SEAc for urban and rural fox data on the population level and the overlap between these two areas.

The relative contribution of the collected food sources to the diet of urban and rural red foxes was estimated with the Bayesian isotope mixing model MixSIAR (Stock & Semmens, 2013). The isotopic signatures of tissues and food sources, fractionation of tissues and variability were used to estimate the isoscape plot and the contribution of the food sources to a mixture. The percentage of concentration of δ^15^N and δ^13^C values in the food sources was included in the analysis. This step is recommended in the case of the large variation of elemental concentrations among the sources (Phillips and Koch, 2002). Rosaceae were excluded from the analysis after prior visualization of the isospace plot, as they lay well outside the source polygon. This could mean either that the source was not consumed by the sampled foxes or that the source is difficult to be found in whiskers. Berries are mainly composed by carbohydrates and therefore they should fuel immediate energy metabolism pathways rather than being incorporated in tissues. For this reason, they are more likely to be found in short‐term samples, such as breath exhaled CO_2_ (Hobson & Stirling, 1997; Hobson, Stirling, & Andriashek, 2009). Additionally, a PERMANOVA test was performed to test pairwise differences between food sources, which could be pooled together for a‐posteriori combining before running the MixSIAR analysis. The Markov Chain Monte Carlo (MCMC) parameter estimates the probability density functions of variables of interest and the entire distribution for each variable. The MCMC parameter chosen to run the MixSIAR was “very long” (chain length of 1,000,000).

To model the isotope ratios (δ ^15^N and δ ^13^C) as a function of the covariates to analyze their potential effects, a linear model was used. Fixed covariates are sex (categorical with two levels), age (categorical with two levels), and Julian day (continuous). Finally, we tested whether the variance of δ^13^C and δ^15^N values differs among the urban and the rural fox population using an F‐Test.

#### 2.3.2 Individual level

We determined the SEAc of the 19 adult urban and 13 adult rural foxes of longitudinal measurements based on stable isotope ratios using Stable Isotope Bayesian Ellipses in R (SIBER package: Jackson et al., 2011). To calculate the degree of individual diet specialization within these two fox populations, we adjusted the mathematical model of Roughgarden (1972), which was discussed by Bolnick et al. (2002) as a possible index for individual specialization. Following that, a foraging specialist is an individual whose dietary niche is narrower than the total niche width (TNW) of the population. The TNW consists of two components, (a) the variation in resource use within individuals (WIC) and (b) the variance between individuals (BIC): TNW = WIC + BIC. To determine the TNW, we used the trophic niche breadth represented by the total area (TA) of the urban and rural population. The WIC is represented by the TA of each individual. When the WIC/TNW ratio approaches 1, all individuals utilize the full range of the population's niche, whereas smaller values indicate an increasing degree of individual diet specialization. To obtain the intra‐ and interspecific variation of the isotopic composition during a one‐way ANOVA a reference value to compare with is needed. Thus, we used that whisker, which covered the largest per mille range for δ^13^C and δ^15^N values.

## 3 RESULTS

### 3.1 Population‐level

After the PERMANOVA test, land snail, land slug, and earthworm were pooled together as they did not differ significantly in isotopic values between each other. As described in the method section, brambles were excluded from further analysis (Appendix C).

**Figure 3:**
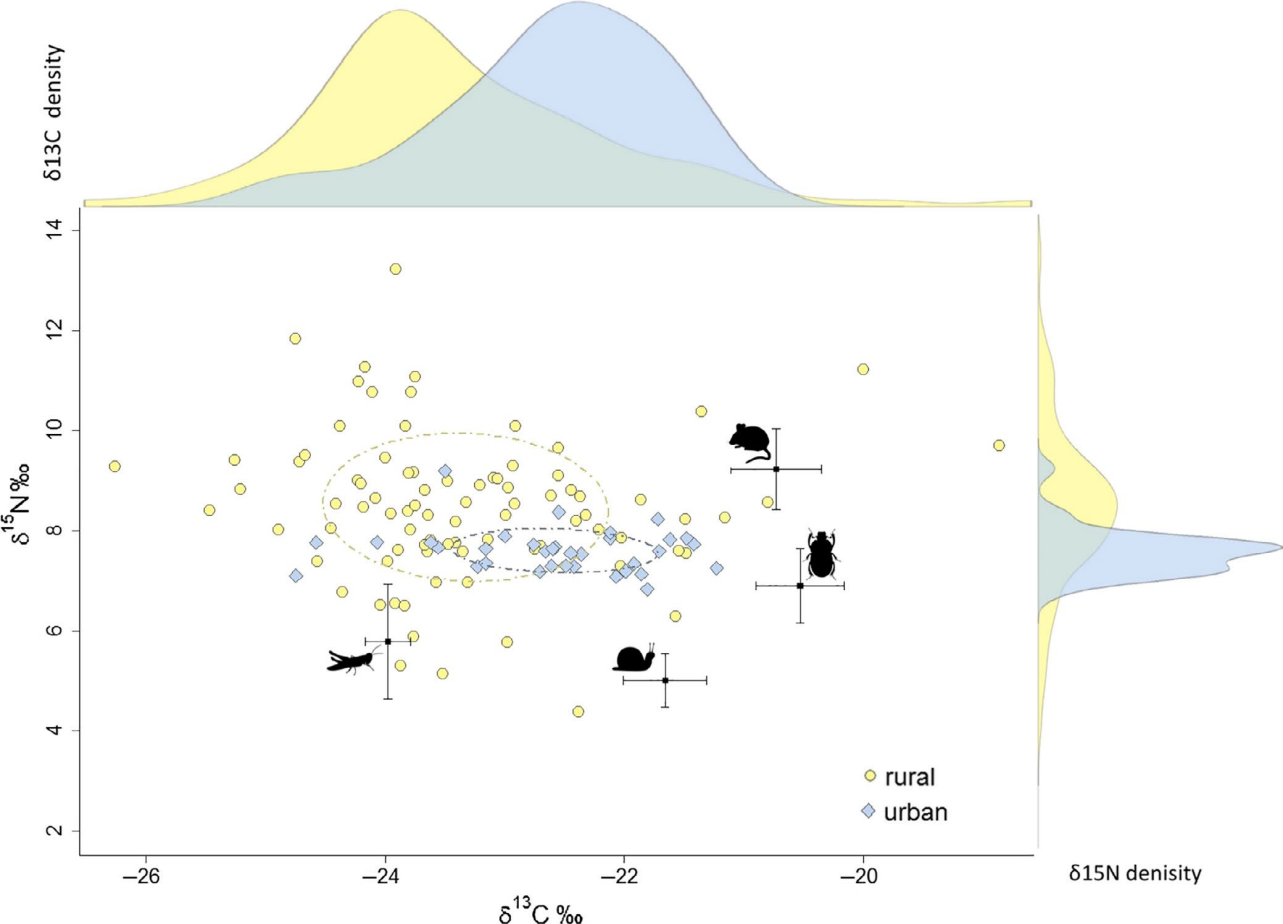
Isospace and density plot for raw δ^13^C and δ^15^N values of urban (blue diamonds) and rural (yellow circles) red foxes whisker samples (*n* = 119) from Berlin and Brandenburg, Germany. Dashed ellipses represent SEAc of the urban (blue) and rural (yellow) fox population. Black dots show trophic corrected mean (±SE bars) δ^13^C and δ^15^N values for the four prey taxa including (1) grasshopper, (2) land slug, land snail, earthworm (pooled together), (3) dor beetle, and (4) house mouse

Isotopic analysis of potential food items of red foxes indicated that the potential isotopic niche was broad for this species (Table 1, Figure 3). The δ^13^C values ranged from −30.1‰ to −24.8‰ and δ^15^N values from 1.7‰ to 6.2‰. We observed an isotopic difference among most food sources (Table 1). The variation in isotopic values of individual food items is relatively small in comparison to the variation between food items.

We tested the distribution of Shannon index values based on land use classes for both, urban (mean = 0.9 ± 0.16) and rural (mean = 0.5 ± 0.27) fox population (see also Appendix A). A Wilcoxon rank‐sum test revealed that the median Shannon index of rural regions is significantly less than the median urban Shannon index (*W* = 267, *p* < .001). Therefore, based on the Shannon index, our urban sites are more diverse on a landscape structure scale than the rural ones.

Our linear model yielded no significant effect of sex, age or Julian day on individual δ^13^C values (adjusted *R* ^2^ = 0.01, *F*(115,3) = 1.405, *p* > .05) or on δ^15^N values (adjusted *R* ^2^ = −0.03, *F*(115,3) = 0.026, *p* > .05). The *F*‐test confirmed a significant difference between the variance of the urban and rural population for δ^13^C values (*F*_(84)_ = 1.908, *p* = .040) as well as δ^15^N values (*F*_(84)_ = 11.394, *p* < .001).

The isotopic compositions of the 34 urban fox whiskers averaged 7.6 ± 0.4‰ for δ^15^N (range 6.8 to 9.2‰) and −22.6 ± 0.9‰ for δ^13^C (range −24.8 to −21.2‰), those of the 85 rural foxes 8.5 ± 1.5‰ for δ^15^N (range 4.4 to 13.2‰) and −23.3 ± 1.2‰ for δ^13^C values (range −26.3 to −18.9‰).

The stable isotope ratios of individual whiskers (*n* = 119, Figure 3) varied largely in their δ^13^C and δ^15^N values. δ^15^N and δ^13^C values of some red foxes fell outside the range of food stable isotope ratios, indicating that foxes might have consumed food resources of high δ^15^N and low δ^13^C values.

A Welch two‐sample *t* test confirmed the significant difference between the means of the urban and rural population for δ^13^C (*t*_(83,479)_ = −3.77, *p* < .001) as well as δ^15^N values (*t*_(111,27)_ = 4.89, *p* < .001). Additionally, the two populations differed in total area (TA) and SEAc indicating different isotopic niches. Rural foxes showed a TA of 36.8‰^2^ and a SEAc of 5.6‰^2^, whereas the urban population had a narrower isotopic niche with a TA of 5.2‰^2^ and a SEAc of 1.2‰^2^. The overlap between the SEAc of the two populations was 0.8‰^2^, consisting of 66.7% of urban and 14.3% of rural SEAc size.

### 3.2 2.2. Individual‐level

The isotopic niche of rural foxes (TA = 3.3‰^2^) was broader than the isotopic niche of urban foxes (TA = 1.7‰^2^), with urban foxes averaging 7.4 ± 0.7‰ for δ^15^N and −22.8 ± 0.9‰ for δ^13^C values and rural foxes averaging 8.3 ± 1.0‰ for δ^15^N and −23.5 ± 1.1‰ for δ^13^C values. SEAc values of individual whiskers ranged from 0.1‰^2^ to 1.8‰^2^ in urban and 0.1‰^2^ to 5.0‰^2^ in rural foxes.

**Figure 4:**
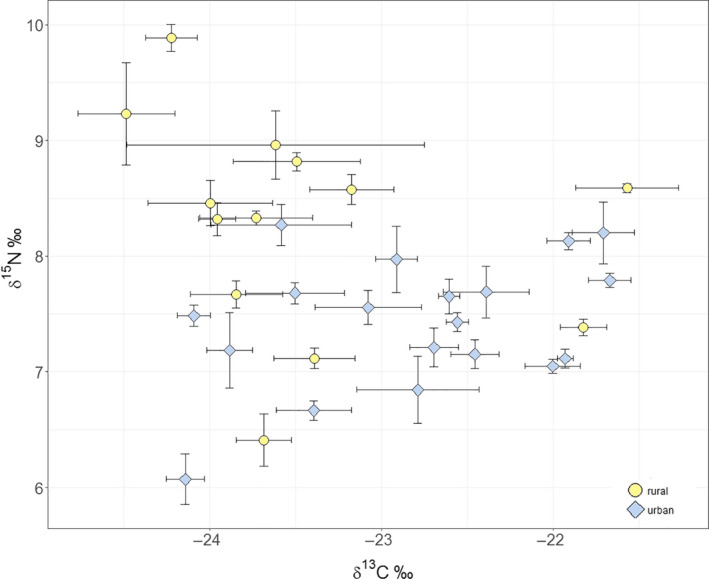
Mean C and *N* values over time of all segments sampled from 19 urban (blue diamonds) and 13 rural (yellow circles) red fox individuals from Berlin and Brandenburg, Germany. Error bars represent the standard deviation *SD*

Rural red foxes differed in the consumption of isotopically different food items with regard to δ^13^C (one‐way ANOVA: *F*_(12)_ = 6.094, *p* < .001) and δ^15^N values (*F*_(12)_ = 24.741, *p* < .001). This pattern was similar for urban foxes for both δ^13^C (*F*_(18)_ = 15.215, *p* < .001) and δ^15^N values (*F*_(18)_ = 9.697, *p* < .001). Looking at the results of the analysis of repeated measurements over time for each individual (Figure 4, Appendix B), the stable isotope values remain relatively similar and show little variance. Foxes in total used a wide range of the available isotopic dietary space, but individuals (apart from a few exceptions) utilized just a relatively small portion of the total range.

**Figure 5:**
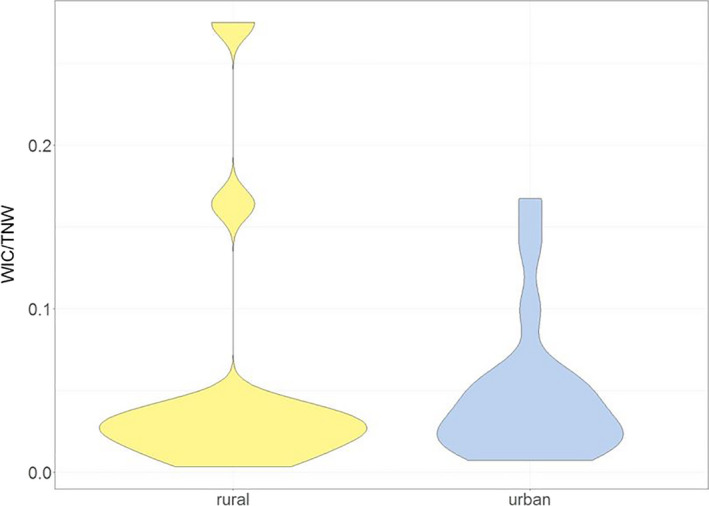
WIC/TNW ratios for 19 urban and 13 rural adult red foxes. The WIC/TNW ratio is a measurement which represents the degree of individual diet specialization within a population. When the ratio approaches 1, all individuals utilize the full range of the population's niche. Since the ratio was very small for both populations (urban and rural) red foxes show a high degree of individual diet specialization

The WIC/TNW ratio as a measure of feeding specialization was very small in both populations (Figure 5). The mean WIC/TNW was 0.05 (±0.08) and 0.05 (±0.04), respectively, for both, rural and urban foxes, indicating a highly specialized resource use at an individual level.

## 4 DISCUSSION

We investigated diet niche width and the feeding tactics of urban and rural red foxes at (I) the population level in space and (II) the individual level over space and time. For this purpose, we used carbon (δ^13^C) and nitrogen (δ^15^N) isotopic signatures of red fox whiskers as well as longitudinal measurements concerning these two isotopes. The diet of red foxes has been studied over decades (e.g., Calisti et al., 1990; Contesse et al., 2004; Díaz‐Ruiz et al., 2013; Englund, 1965; Harris, 1981; Leckie et al., 1998; Macdonald, 2011), but just a few studies have investigated the variation within the fox population and thereof only sporadically on a fine temporal scale (Molsher, Gifford, & McIlroy, 2000). To our knowledge, our analysis is the first empirical investigation allowing conclusions about potential individual dietary specialization which compares feeding tactics of individuals between urban and rural foxes over time. Thus, our study provides a more detailed insight into the foraging strategies of this successful predator in human‐dominated landscapes. This is important to understand the complex interrelationships associated with increasing urbanization. Stomach content analysis and the investigation of feces composition have been the primary methods in previous studies on red fox feeding ecology (e.g., Baker, Furlong, Southern, & Harris, 2006; Calisti et al., 1990; Cavallini & Volpi, 1995; Dell’Arte, Laaksonen, Norrdahl, & Korpimäki, 2007; Doncaster et al., 1990; Englund, 1965; Goldyn, Hromada, Surmacki, & Tryjanowski, 2003; Harris, 1981; Jedrzejewski & Jedrzejewska, 1992; Kolb & Hewson, 1979; Leckie et al., 1998; Panek, 2013; Panek & Budny, 2017). Those traditional ways of analyzing diets often fail to adequately reveal temporal patterns and pseudoreplication is a recurring problem (Dalerum & Angerbjorn, 2005; Darimont & Reimchen, 2002; Deb, 1997). Additionally, results are biased because of sampling methods and physiology of digestion (Cavallini & Volpi, 1995; Meckstroth, Miles, & Chandra, 2007) and conventional methods such as fecal sample analysis are usually only a snapshot and in this case, do not provide longitudinal data over time because sampling repeatedly feces of a specific individual in the wild is challenging, time‐consuming and may require additional genetic testing. However, individual differences in resource use are crucial for understanding food webs, disease transmission, and for effective wildlife management. Stable isotope analysis is the means of choice when it comes to deciphering the degree of food specialization since isotopes reflect average dietary records and represent assimilated rather than ingested foods, thus bypassing some of the shortcomings of stomach content or feces analysis (Crawford, Mcdonald, & Bearhop, 2008; Dalerum & Angerbjorn, 2005; DeNiro & Epstein, 1978, 1981). This kind of method offers a broader temporal resolution of dietary differences. It is also robust to short‐term variations because of food availability, foraging opportunity or preference and stable isotope ratios of red fox tissues should reflect food items within their home range (Lavin et al., 2003).Nevertheless, stable isotope analyses also have their limitations. Thus, we cannot make an accurate statement about the food that is eaten. Since the metabolic turnover time (time of food intake to time of representation of the diet in organ of interest) for red fox vibrissae has not yet been investigated, we cannot make precise statements about the timing of food intake, which would be speculative to compare with potential seasonal food supply. Although nitrogen isotope signatures (δ^15^N) provide powerful measures of the trophic positions of individuals and populations, normally you have to apply baseline corrections to account for spatial variation (Woodcock et al., 2012). This is not meaningful for our study design. Nitzsche, Verch, Premke, Gessler, and Kayler (2016) have shown small‐scale variations in plant, soil, and sediment nitrogen isotope signatures even within the area of a typical red fox home range. Although foxes are locally bound when they have established their territory, they are nevertheless very mobile and generalist feeder within its range. Therefore, an effort to investigate spatial variability at the base level by collecting additional potentially suitable soil or plant samples is pointless. In contrast, we have collected potential food resources of different trophic levels at different locations within the study area along the urban‐rural gradient. This gives us at least an impression of the variability of the stable isotope signatures of some typical food items and therefore the basis to correctly interpret the results of the red fox data. Although the food items were collected both in the highly urbanized city center of Berlin and in rural areas, the variance of isotopic values of the individual resources was small. Thus, a large variation in isotope values of the red foxes cannot be attributed to a large variance of single food items and can, therefore, be interpreted as variability in diet composition or food selection. Besides, the collection of potential food items along the rural‐urban gradient reflects a general availability of these resources for urban and rural foxes, even though we cannot make any statements about the food density.

### 4.1 Population‐level

First of all, neither in the urban nor the rural population did we observe differences between sexes or age‐classes on dietary choice. This confirms other studies showing that offspring consume the same food as adults (Kolb & Hewson, 1979; Weber, 1996) but contradicts (Panzacchi, Linnell, Serrao, et al., 2008) who demonstrated a significantly higher amount of large prey types fed to cubs in rural regions. A bigger prey size maximizes the energetic benefit and reduces the relative costs connected with returning to the den and feeding their offspring. Juveniles develop their hunting skills at the age of approximately 6 months (Harris & Trewhella, 1988) and they focus on easy prey at the beginning. Since in our case "juveniles" are all animals <1 year, our study design does not allow reconstructing such differences. In contrast to our results, Kidawa and Kowalczyk (2011) revealed sex‐related diet preferences within adult red foxes. These differences are probably connected to the breeding period. A further explanation is that reproductive females probably monopolize superior food patches at the expense of younger nonreproductive animals.

In total, isotopic δ^13^C and δ^15^N values of rural and urban red foxes (*n* = 119) showed a broad range spanning multiple trophic levels and included all of the food items we examined specifically (Figure 3). This corroborates current knowledge that the red fox is a food generalist (Doncaster et al., 1990; Englund, 1965; Jędrzejewski & Jędrzejewska, 1992). For a brief overview, a review of 55 studies from the Iberian Peninsula on red fox diet found a biogeographical relation between the consumption of lagomorphs and invertebrates as well as the intake of small mammals and fruits/seeds on the other hand (Díaz‐Ruiz et al., 2013). Thus, the red fox showed that variation in feeding habits depending on environmental factors which determine the availability of their main food. Results of studies on red fox diet in agricultural landscapes yielded a diet mainly based on rodents and game birds (e.g., Jankowiak, Antczak, & Tryjanowski, 2008; Leckie et al., 1998). In contrast to that, food of vegetable origin (fruits and seeds) is important in the diet of red foxes from the Mediterranean coastal area (Calisti et al., 1990). In general, red fox diet seems to be highly diverse. Nevertheless, our whisker samples of the rural population showed higher variability in isotopic signature and therefore denote a broader isotopic dietary niche than urban foxes although overlapping with foxes of urban areas (Figure 3). Bearhop et al. (2004) predicted that populations feeding on a wide range of prey species will exhibit wider variation in their tissue isotopic signatures, and populations, where individuals consume prey over a broad spectrum of trophic levels, will tend to show more isotopic variance than those which feed on the same number of prey species, but same trophic level. Accordingly, it is likely that the rural population consumes a wider range of different prey species spanning multiple trophic levels than the urban one.

However, an urban environment is a complex mosaic of different elements on a relatively small spatial scale, although consisting of much sealed area (high imperviousness value). Therefore, the mean Shannon diversity index of urban areas is higher than for rural regions (see Appendix A). Surprisingly, although thus urban areas are more heterogeneous on a habitat scale and should offer also a more diverse food spectrum for generalist species (Tews et al., 2004), the urban foxes don't feed as varied or broad as its rural conspecifics. A recent review using data from 66 studies in 17 European countries revealed that dietary breadth of red foxes increases in areas with high human impact (Soe et al., 2017). This may be since we used the degree of imperviousness as an explanatory variable, whereas colleagues used the human footprint index to determine human impact. However, this also includes agricultural areas that are categorized as rural in our study, although they are dominated by humans. Nevertheless, rural areas defined as an area having less than 25% sealed surface comprise more diverse habitat types in total, which together offer a wide range of prey species and food sources. Cities, on the other hand, provide an elementary and instantly available food source for urban foxes (Contesse et al., 2004), potentially explaining the narrower isotopic niche: anthropogenic food.

It is difficult to detect anthropogenic food items using traditional methods (e.g., macroscopic inspection of scats), as processed food usually does not contain identifiable, indigestible material such as exoskeletons, bones, feathers or hair (Meckstroth et al., 2007). However, Newsome et al. (2010) were able to show that urban kit foxes (*Vulpes macrotis mutica*), had significantly higher δ^13^C values (difference in mean = 2.4‰) and lower δ^15^N values (difference in mean = 2.7‰) than nonurban individuals and isotopic values similar to human residents. Based on their findings they suggested a shared (anthropogenic) food source and similarities in their diet. Meaty anthropogenic food contains a noticeable amount of corn because livestock reared for meat production is often fed a corn‐based diet. Food crops like maize as well as sugar cane, millet, and sorghum are typical C_4_ plants, which differ in their δ^13^C values (−12 to −14‰) from C_3_ plants (−22 to −29‰) (Craig, 1953; Farquhar, Ehleringer, & Hubick, 1989). Urban wildlife that feeds on anthropogenic sources shows slightly higher δ^13^C values, because of the direct or indirect influence of C_4_ plants described before. As anthropogenic food also consists of food items of low trophic level (e.g., pastries, fruits, and vegetables), individuals feeding on anthropogenic food sources also have lower δ^15^N values than individuals which focus on natural prey animals (Lavin et al., 2003; Murray et al., 2015; Newsome et al., 2015). All this leads to the conclusion that also urban red foxes of our study area have established anthropogenic food in their diet. This corresponds to the diet of urban foxes in other cities (Doncaster et al., 1990; Harris, 1981; Saunders et al., 1993). Red foxes in Zurich, for example, had filled more than half of their stomachs with anthropogenic food whereas the proportion of anthropogenic food increased toward the city center (Contesse et al., 2004). Also, the estimation of a lifetime diet of red foxes in Alaska suggests a consistent use of anthropogenic food and this access is directly associated with their expansion and successful establishment (Gallant, Slough, Reid, & Berteaux, 2012; Savory, Hunter, Wooller, & O’Brien, 2014). In general, urban wildlife may prefer anthropogenic food over natural sources because of constant availability, predictability, and lower foraging costs (Contesse et al., 2004; Weiser & Powell, 2010). A higher amount of anthropogenic food could potentially lead to increased human‐wildlife encounter rates and therefore foster conflicts (Murray et al., 2015; Panek & Budny, 2017).

### 4.2 Individual‐level

Our results of longitudinal data on individual‐level strengthened our previous findings. Since the TA of rural foxes is bigger in comparison with urban individuals, rural foxes cover a broader isotopic niche and therefore dietary spectrum. Again, mean δ^15^N value is smaller and the mean δ^13^C value is bigger for urban foxes which confirm the difference in foraging behavior. Focusing on the SEAc values of individual whiskers, it can be seen that urban individuals have an even narrower isotopic dietary niche than individuals from rural areas.

At any time or location, the realized niche of a population represents the sum of all prey consumed by individuals belonging to this population (Bolnick et al., 2002). Accordingly, there is a clear link between feeding tactic at the individual level and trophic interactions defined at the population or species level. Our results show that the stable isotope values of red foxes varied among and between urban and rural individuals (Figure 4). Therefore, individuals feed on different prey items or have a different diet composition compared with conspecifics. The variability of the diet over time is represented by the standard deviation. Since the degree of variability was low, likely, the relative proportion of food items in the diets did not vary largely and individuals focused on the same diet. Moreover, urban as well as rural foxes may have consumed only a small proportion of potentially available isotopic dietary niche. Interestingly, the diet of a particular individual remained chiefly constant over the time reflected by whisker length (approximately 2 months, see Appendix B), since the variance of δ^13^C and δ^15^N values of the different whisker increments is low. Moreover, WIC/TNW ratio is very low in both populations (Figure 5) and therefore the individual dietary niche is substantially narrower than the total niche with the population. By definition (Bolnick et al., 2002) and in addition to all other previous results of longitudinal analysis, generalistic fox populations consist of individual food specialists at least over medium periods (here approximately 2 months) in our study area.

Although previous studies have already shown that foxes are generalists at the species level, their diet may vary regionally and may also be highly specialized at the population level (Dalerum & Angerbjorn, 2005; Díaz‐Ruiz et al., 2013; Panzacchi, Linnell, Odden, Odden, & Andersen, 2008; Soe et al., 2017), we were able to show that foxes can also be food specialists at the individual level. However, our method only allows us to make this statement for about 2 months. It should, therefore, be discussed whether this specialization is possibly due to limited food availability at the respective local sampling locations or corresponding seasons. Fox samples were collected over a large area and long distances, including a wide variety of habitats. It can be assumed that the food supply in the forests certainly differs from agricultural landscapes or in the vicinity of settlements and that this has led to the (inter‐individual) variance of isotope values at population and individual level. Although we have not studied food availability, in particular, it can also be assumed that more diverse habitats (measured by the Shannon index in this study) provide a more diverse food supply for opportunists (Tews et al., 2004). The situation is similar to the seasonal availability of food. Our sampling regime covers different seasons and especially in seasons like autumn, a wide variety of food sources such as fruits, insects, and small vertebrates are potentially available in our region (Drygala et al., 2013). If the season influenced the diet, we would expect differences in diet and degree of specialization between foxes from seasons with a varied diet (e.g., autumn) and limited availability of food resources (e.g., winter). This is not supported by our results. Furthermore, the linear model has not been able to demonstrate any effect of time in the year on the distribution of isotope values. If the patterns found were due to food availability, then foxes in very diverse habitats or with extensive food supply spanning several trophic levels would show a high variance in their individual isotope values over time. This was not the case in our investigation. It can thus be assumed that foxes are generally specialized in a small number of food resources, or use different food resources but always integrate them into their diet in very similar proportions. Differences in diet composition among conspecifics and dietary specialization have been documented across a broad range of taxonomic groups and habitats (reviewed in Bolnick et al., 2002) and such variation at the individual level is increasingly recognized as an important component of diversity in trophic interactions. Furthermore, the extent to which population‐level dietary patterns are determined by the composition of similar or very different individuals has potentially important implications to behavioral and evolutionary ecology, ecosystem dynamics and conservation efforts (Tinker, Bentall, & Estes, 2008). However, the reasons leading to individual specialization can be very different. For example, a wide isotopic dietary niche of the population with heterogeneous use of the resources, combined with narrow individual niche widths, reduces intraspecific competition. If food specialization would only reflect food availability and thus the habitat, structurally very similar habitats with the same food disposability should result in overlapping isotopic food niches of different individuals. An avoidance of intraspecific competition could be necessary in case of decrease of habitat quality or quantity or an increase in population density (Araújo et al., 2011). Therefore, competitive and dominant individuals may monopolize territories of high quality and subordinate individuals will then be forced to resort to alternatives of lower quality (Morse, 1974). In contrast, a very broad niche may increase interspecific competition (Vellend, 2016) due to diet overlap with other species. It also reduces the impact of this overlap because only a subset of individuals in each species is affected when individuals of a generalist species feeding specialized. On the other hand, depending on resource availability, food generalists may nevertheless be factual individual foraging specialists because of different environmental and social factors such as social status, territory location, or even trade‐offs constraining the ability of individuals to forage. The variation between conspecifics in resource use can also reflect intra‐population variation based on individual traits such as resource‐specific preference and efficiency (Bolnick et al., 2002). Therefore, the success of foraging behavior and prey capture bases on learning and experience; insufficiencies will restrict the handicapped individual to a limited range of prey (Kato et al., 2000). Individuals of (food‐)generalist species often use the same resources if only this resource is available or a particularly high‐quality food source is very abundant and easily accessible (Robinson & Wilson, 1998). Consequently, complex interactions of different factors affect individual resource use and feeding tactics.

### 4.3 General discussion

Different foraging and feeding habits can alter a cascade of direct and indirect effects. Identifying intraspecific trait variation, in our case, the different foraging niches of conspecifics are of enormous importance to understand ecological dynamics, because it will alter population densities, transient dynamics, and persistence (Bolnick et al., 2011). At an individual level, the utilization of anthropogenic food subsidies is often predictable in space and time and can increase fitness (Oro, Genovart, Tavecchia, Fowler, & Martínez‐Abraín, 2013). In contrast, different diets may incur different risks, for example, differences in parasite load of certain food items. Food generalists are more likely to encounter multiple parasite species because they consume various intermediate hosts. The exposure to a wider variety of different parasites at low frequencies may be worse than high exposure to a limited spectrum of parasites when there are trade‐offs in the immune response to several parasite species (Curtis, Bérubé, & Stenzel, 1995). On the other hand, information about the diet at the individual level of food specialists within a generalistic population could be important to estimate the spread of specific parasite species within the host species. In this case, looking only at the population level would lead to a wrong impression. Is a diet connected to a specific habitat, there could be also a difference in predation or mortality risk (Durell, 2000) depending on the habitat. Moreover, individual feeding tactics also affect population dynamics as well as communities and ecosystems. They alter food webs, within‐community competition, and predator‐prey dynamics because food generalists have an impact on a diverse prey assemblage and food specialists influence only a limited assortment. Furthermore, they also promote the invasion of non‐native species and increase human‐wildlife conflicts (Oro et al., 2013). Identifying differences in feeding tactics on an individual level has also increasingly been recognized as an important part of population ecology because it helps to create individual‐based models for a mechanistic understanding of processes and patterns (Bolnick et al., 2003). The investigation of foraging tactics is therefore of particular importance, because only then can we correctly interpret occurring patterns and relationships and ultimately develop appropriate strategies to promote biodiversity in cities and minimize human‐wildlife conflicts.

## CONFLICT OF INTEREST

The authors declare that the research was conducted in the absence of any commercial or financial relationships that could be construed as a potential conflict of interest.

## AUTHOR CONTRIBUTION

**Carolin Scholz:** Conceptualization (equal); Data curation (supporting); Formal analysis (equal); Project administration (lead); Visualization (lead); Writing‐original draft (lead); Writing‐review & editing (equal). **Jasmin Firozpoor:** Conceptualization (supporting); Formal analysis (equal); Investigation (equal); Writing‐original draft (supporting); Writing‐review & editing (equal). **Stephanie Kramer‐Schadt:** Conceptualization (equal); Supervision (lead); Writing‐review & editing (equal). **Pierre Gras:** Formal analysis (supporting); Writing‐review & editing (equal). **Sophia E. Kimmig:** Data curation (supporting); Writing‐review & editing (equal). **Christoph Schulze:** Data curation (lead); Writing‐review & editing (equal). **Christian C. Voigt:** Conceptualization (equal); Methodology (lead); Resources (supporting); Supervision (lead); Writing‐review & editing (equal). **Sylvia Ortmann:** Conceptualization (equal); Funding acquisition (lead); Resources (lead); Supervision (lead); Writing‐review & editing (equal).

## Data Availability

The data that support the findings of this study are openly available in Dryad at https://doi.org/10.5061/dryad.6t1g1jww8.
